# Astragalus Polysaccharide Protects Astrocytes from Being Infected by HSV-1 through TLR3/NF-*κ*B Signaling Pathway

**DOI:** 10.1155/2014/285356

**Published:** 2014-06-26

**Authors:** Lihong Shi, Fengling Yin, Xiangui Xin, Shumei Mao, Pingping Hu, Chunzhen Zhao, Xiuning Sun

**Affiliations:** ^1^Department of Pharmacology, Weifang Medical University, 7166 Baotong West Street, Weifang 261053, China; ^2^Hospital of Weifang Medical University, 7166 Baotong West Street, Weifang 261053, China; ^3^The Affiliated Hospital of Shandong Traditional Chinese Medicine Junior College, 508 Binhai East Road, Yantai 264199, China; ^4^Department of Parasitology, Weifang Medical University, 7166 Baotong West Street, Weifang 261053, China

## Abstract

Astragalus polysaccharide (APS) is the most immunoreactive substance in Astragalus. APS can regulate the body's immunity and is widely used in many immune related diseases. However, till now, there is little information about its contribution to the protection of astrocytes infected by virus. Toll-like receptor 3 (TLR3) is a key component of the innate immune system and has the ability to detect virus infection and trigger host defence responses. This study was undertaken to elucidate the protective effect of APS on herpes simplex virus (HSV-1) infected astrocytes and the underlying mechanisms. The results showed that APS protected the astrocytes from HSV-1 induced proliferation inhibition along with increasing expression of tumor necrosis factor-*α* (TNF-*α*) and interleukin-6 (IL-6) markedly. Moreover, APS significantly promoted the expression of Toll-like receptor 3 (TLR3) and the activation of nuclear factor-*κ*B (NF-*κ*B) in astrocytes. In addition, while astrocytes were pretreated with TLR3 antibody before adding HSV-1 and APS, the expression of TLR3, TNF-*α*, and IL-6 and the activation of NF-*κ*B decreased sharply. These results indicate that APS can protect astrocytes by promoting immunological function provoked by HSV-1 through TLR3/NF-*κ*B pathway.

## 1. Introduction

Herpes simplex encephalitis (HSE) is the most common sporadic and nonepidemic viral encephalitis in both children and adults. Untreated HSE can result in prolonged neuroinflammation and compromised brain function or death [1–4]. Furthermore, the majority of treated patients would suffer various degrees of sequela despite the improvements in diagnosis and therapy [5]. HSV-1 infection of the brain results in devastating necrotizing encephalitis. Murine model of herpes simplex encephalitis showed that HSV-1 infection triggered a robust immune response including infiltration of leukocytes, production of proinflammatory mediators, activation of resident microglial cells, and focal tissue damage.

Toll-like receptor is an important class of immune recognition receptors that can recognize pathogens molecules, activate the innate immune response and invoke the releasing of cytokines, and initiate adaptive immunity [6, 7]. As an important member of the Toll-like receptor family, TLR3 can recognize double-stranded RNA (dsRNA) of viruses and induce related signal pathway to play important role in host defense against viruses [8, 9]. It is a remarkable fact that TLR3 is vital for natural immunity to HSV-1 in the central nervous system [10, 11].

Astrocytes are the most abundant cells in the central nervous system (CNS). The main function of astrocytes is to provide nutrition and support. In addition, astrocytes can secrete cytokines and neurotrophic factors to regulate the immune function [12, 13]. When mice embryos were infected by HSV-1, neuronal injury and necrosis accompanied by pathological changes of astrocytes would occur [14]. Recently, Furr et al. reported that astrocytes increased expression of DNA-dependent activator of interferon-regulatory factors (DAI) as an important innate immune mechanism underlying the rapid and potentially lethal inflammation associated with HSV-1 infection [15]. Our previous studies revealed that when astrocytes were infected by HSV-1, NF-*κ*B was activated through Toll-like receptor 3 (TLR3) and then the activated NF-*κ*B would translocate from the cytoplasm to the nucleus so as to promote the production of TNF-*α* and IL-6 to play antiviral roles [16]. These results demonstrate that astrocytes play significant roles in the inflammatory responses of resident CNS cells to HSV-1 challenge.

Astragalus is a traditional Chinese medicine which contains polysaccharides, saponins, flavonoids, amino acids, linoleic acid, alkaloids, and so forth. Astragalus polysaccharide (APS) is the most immunoreactive substance in Astragalus which can regulate the body immunity. APS has been identified as a class of macromolecules that can profoundly affect the immune system and is widely used as an immune adjuvant in China. APS can induce the expression of surface antigens on lymphocytes, promote the production of antibodies, affect the secretion of cytokines, and even stimulate cell proliferation [17, 18]. Previous studies proved the effective immunostimulatory roles of APS against various viruses [17, 19, 20].

In this paper, based on our previous research, the antiviral effect of APS on the HSV-1 infected astrocytes was investigated. Furthermore, the immunoregulatory effect and the possible immunization mechanisms of APS were evaluated.

## 2. Materials and Methods

### 2.1. Laboratory Animals, Cells, and Virus

The BALB/c mice were purchased from Medicine Animal Center of Shandong University. HSV-1 SM44 strain was kept in Central Laboratory of Weifang Medical University at −80°C. The rabbit anti-mouse antibody TLR3, NF-*κ*B, and *β*-actin were from Invitrogen; Nuclear and Cytoplasmic Protein Extraction Kit were from Beyotime Institute of Biotechnology; MTT kit was purchased from Sigma; 96T enzyme-linked immunosorbent assay (ELISA) kit was purchased from ADL; APS was purchased from Tianjin Sino Pharmaceutical Company; batch number is 120102.

This research was conducted in strict accordance with the recommendations in the Guide for the Care and Use of Laboratory Animals of the National Institutes of Health. Surgery was performed under sodium pentobarbital anesthesia; all efforts were made to minimize suffering. The protocol was approved by the Committee on the Ethics of Animal Experiments of Weifang Medical University (number 2012-056).

### 2.2. HSV-1 Multiplication and Tittering

The HSV-1 strain SM44 propagated in Vero cells; the supernatant was collected when cytopathic effect (CPE) was in confluence to 75%. By freezing in −80°C and thawing for three times, HSV-1 particles were released. By centrifuging for 8 min with 1200 rpm, the supernatant was collected as inoculum and stocked in freezer. All of the HSV-1 samples used in this study were of the same batch. In following research, final concentration of HSV-1 : TCID_50_ was 10^−6^ mL^−1^.

### 2.3. Astrocytes Culture and Purification

Newborn BALB/c mice in day 1 to day 3 were taken and cells culture and purification were performed according to McNaught's protocol [21]. Take the 3rd generation of astrocytes for experimental research according to our team's protocol [16].

### 2.4. MTT Cell Proliferation Assay, Calculating the Median Effective Concentration (EC_50_) of APS

Astrocytes (1 × 10^6^ mL^−1^) were cultured in DMEM/F12 media in the presence of HSV-1 (final TCID_50_: 10^−6^ mL^−1^) with APS (0, 50, 100, 200, and 300 *μ*g mL^−1^), 5 repeating groups for each concentration. 20 *μ*L MTT solution (5 mg mL^−1^) was added to cell media at various time points (6, 12, 18, and 24 h) after the addition of HSV-1 and APS, incubated for 4 h, and then supernatants were gently removed and 200 *μ*L dimethyl sulfoxide was added into each pore. After 10 minutes' vibration, the absorbance (OD value) was measured at a wavelength of 492 nm by enzyme-linked immunosorbent detector. OD value is proportional to the proliferation of living cells and is an indicator of cell proliferation.

The mean and standard deviation of OD values for each concentration of APS at every action time point were calculated. The results showed that cell proliferation is the most exuberant at 12 h time point for every concentration of APS, so 12 h is the detection time point in the following assays. EC_50_ of APS was 120 *μ*g mL^−1^, so in the following research, the final concentration of APS was 120 *μ*g mL^−1^. The formula to calculated EC_50_ is

(1) effect%=ODdrug−ODmodelODcontrol−ODmodel×100%.

### 2.5. Grouping and Detecting the Effect of APS on Astrocytes Proliferation by MTT Assay

Astrocytes were seeded at density of 1 × 10^6^ mL^−1^ into 24-well plates; four groups were set up according to the different intervention conditions: HSV-1 group, HSV-1 + APS group, TLR3 antibody + HSV-1 + APS group, and blank control group. In HSV-1 groups, all flask cells were inoculated with viral suspension (final TCID_50_: 10^−6^ mL^−1^); in HSV-1 + APS group, all flask cells were inoculated with viral suspension (final TCID_50_: 10^−6^ mL^−1^) and APS (final concentration: 120 *μ*g mL^−1^); in TLR3 antibody + HSV-1 + APS group, TLR3 antibody (final concentration: 10 *μ*g mL^−1^) was used to pretreat cells for 30 min, and then viral suspension (final TCID_50_: 10^−^^6^ mL^−^^1^) and APS (final concentration: 120 *μ*g mL^−^^1^) were added to cells; in blank control group, the same volume of cell culture medium as the aforementioned groups was added into each flask cells. For each group, 3 repeating experiments were performed. The proliferation rate of cells was assayed by MTT after culturing for 12 h.

### 2.6. Determination of the Levels of TNF-*α* and IL-6 by ELISA

Astrocytes were seeded at density of 1 × 10^6^ mL^−1^ into 12 flasks (25 cm^2^). Grouping and treatment were performed as previously mentioned. Supernatants were collected and filtered. TNF-*α* and IL-6 in the supernatant were measured by ELISA. The absorbance (OD value) was determined using a microplate reader at a wavelength of 450 nm. For each sample, the measurement was repeated 3 times and the average concentration of TNF-*α* and IL-6 was set as the final result.

### 2.7. Detection of TLR3 Protein in Cells and NF-*κ*B Protein in Cell Nuclei by Western Blot

In brief, total protein and nuclear protein were extracted following the reagent company's instruction. Protein samples were separated by SDS-PAGE, transferred to polyvinylidene difluoride membranes, immunoblotted using the appropriate primary and the HRP conjugated secondary antibodies, and visualized by using enhanced chemiluminescence reagents ECL. Anti-*β*-actin or LMNB1 monoclonal antibody was used as loading control. The intensities of bands in Western blots were quantified by densitometry analysis using AlphaImager HP (Alpha Innotech, USA) and NIH Image J software (Rockville, MD, USA). Western blot data shown in the paper are representatives of three independent experiments.

### 2.8. Statistical Analysis

All data were analyzed using the SPSS 13.0 statistical software. Values were expressed as mean ± standard deviation $\left(\overset{-}{X}\pm S\right)$. The significance of differences between groups was determined using the one-way ANOVA. Statistical significance was accepted for *P* values <0.05.

## 3. Result

**Figure 1:**
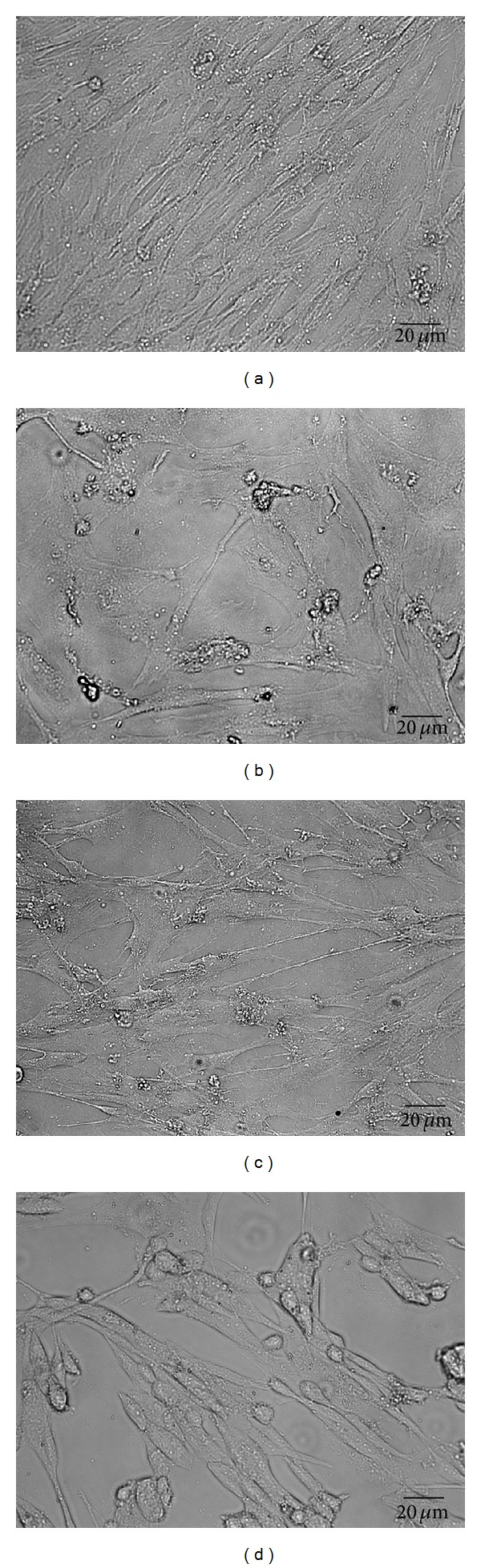
Effect of APS on the growth and proliferation of astrocytes. (a) Blank control group: the astrocytes grew in good condition with active proliferation. (b) HSV-1 group: 12 h after infection with HSV-1, the proliferation of astrocytes was significantly inhibited and the infected astrocytes' bodies were gradually swollen into round and giant appearance. (c) HSV-1 + APS group: the proliferation inhibitory effect of HSV-1 could be suppressed by APS, and most astrocytes grew in good conditiosn. (d) TLR3 antibody + HSV-1 + APS group: when astrocytes were pretreated with TLR3 antibody, the proliferation of astrocytes reduced markedly, and some infected astrocytes' bodies were swollen into round appearance. Scale bar: 20 *μ*m.

APS promotes the growth and proliferation of astrocytes infected by HSV-1. Observation under microscope showed that, in the blank control group, the uninfected astrocytes were in thin and flat appearance with good refraction and grew in good condition with active proliferation (Figure 1(a)); in the HSV-1 group, the proliferation of astrocytes was significantly inhibited and the infected astrocytes' bodies were gradually swollen into round and giant appearance (Figure 1(b)); the inhibited proliferation of astrocytes infected by HSV-1 could be rescued by APS apparently in HSV-1 + APS group (Figure 1(c)); when astrocytes were pretreated with TLR3 antibody and then exposed to HSV-1 and APS concurrently, the proliferation of astrocytes reduced markedly compared with the HSV-1 + APS group (Figure 1(d)).

**Figure 2:**
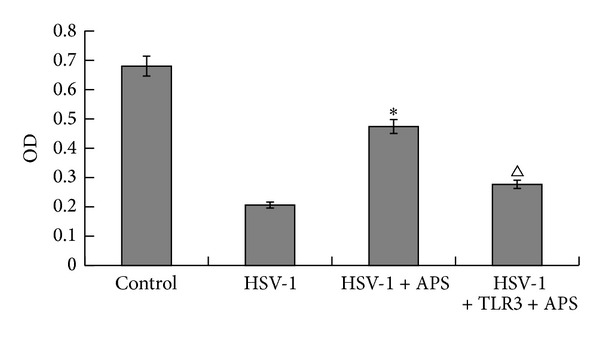
Astrocytes proliferation detected by MTT. *P < 0.01 versus the HSV-1 group. ^Δ^ *P* < 0.05 versus the TLR3 antibody + HSV-1 + APS group. *n* = 3.

MTT analysis (Figure 2) showed that when astrocytes were exposed to HSV-1, the proliferation of astrocytes was significantly inhibited compared to the blank control group. The inhibited proliferation of astrocytes infected by HSV-1 could be rescued by APS apparently in the HSV-1 + APS group. In the presence of APS, the proliferation of astrocytes increased to some extent and the OD value of HSV-1 + APS group was greater than that of the HSV-1 group (*P* < 0.01), which suggests that APS can protect astrocytes from HSV-1 induced proliferation inhibition. Interestingly, when astrocytes were pretreated with TLR3 antibody before adding HSV-1 and APS, the proliferation of astrocytes decreased markedly when compared to the HSV-1 + APS group (*P* < 0.05). This result indicates that the protective effect of APS against HSV-1 infection may be associated with TLR3 pathway.

**Figure 3:**
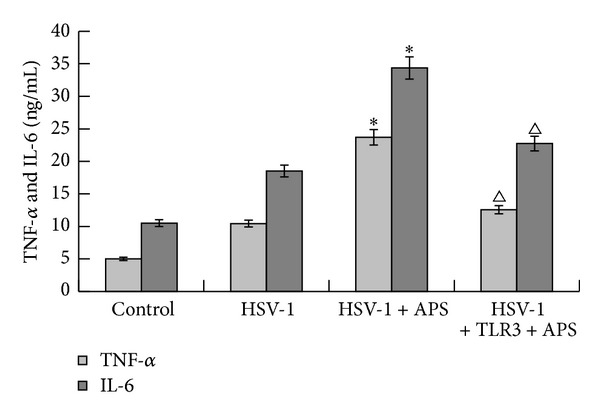
Concentration of TNF-*α* and IL-6 (ng mL^−1^) in cell culture medium detected by ELISA. *P < 0.01 versus the HSV-1 group. ^Δ^ *P* < 0.05 versus the TLR3 antibody + HSV-1 + APS group. *n* = 3.

Secretion levels of TNF-*α* and IL-6 in culture supernatant were detected by ELISA (Figure 3). The concentrations of TNF-*α* and IL-6 were very low in culture supernatant of the blank control group, whereas in culture supernatant of the HSV-1 group, the concentrations of both TNF-*α* and IL-6 increased obviously (*P* < 0.01). In the presence of APS, HSV-1 infected astrocytes expressed higher amount of TNF-*α* and IL-6 than that of the HSV-1 group. Remarkably, pretreatment of astrocytes with TLR3 antibody decreased the expression of TNF-*α* and IL-6 in the TLR3 antibody + HSV-1 + APS group (*P* < 0.05).

**Figure 4:**
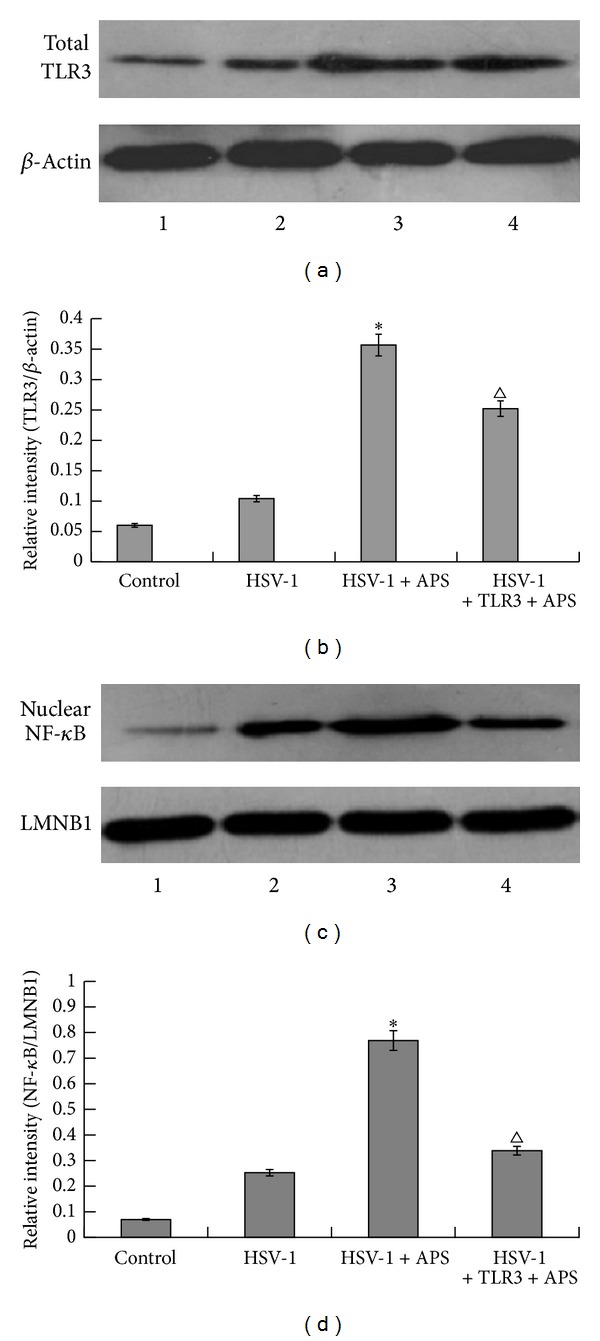
Western blot analysis of expression levels of total TLR3 and nuclear NF-*κ*B (activated NF-*κ*B). (a) Expression levels of total TLR3 protein in blank control group, HSV-1 group, HSV-1 + APS group, and TLR3 antibody + HSV-1 + APS group. *β*-Actin was used as a loading control. (b) The relative intensity of total TLR3 protein as analyzed by Western blot. (c) Expression levels of nuclear NF-*κ*B protein (activated NF-*κ*B) in blank control group, HSV-1 group, HSV-1 + APS group, and TLR3 antibody + HSV-1 + APS group. LMNB1 was used as a loading control. (d) The relative intensity of nuclear NF-*κ*B protein (activated NF-*κ*B) as analyzed by Western blot. *P < 0.01 versus the HSV-1 group. ^Δ^ *P* < 0.05 versus the TLR3 antibody + HSV-1 + APS group. The results of Western blot were from a representative of at least three repeated experiments. *n* = 3.

Western blot analysis showed that the expression level of TLR3 and the activation of NF-*κ*B were low in the control group (Figure 4). In the HSV-1 group, after being exposed to HSV-1 for 12 h, astrocytes expressed higher level of TLR3 than the control group. At the same time, HSV-1 infection induced the activation of NF-*κ*B significantly. Moreover, in the presence of APS, the expression level of TLR3 and the activation level of NF-*κ*B were obviously higher than those of the HSV-1 group. However, when astrocytes were pretreated with TLR3 antibody before addition of HSV-1 and APS, astrocytes reduced the expression of TLR3 and the activation of NF-*κ*B significantly. Considering the different secretion levels of TNF-*α* and IL-6 in different groups, these results indicate that APS may activate NF-*κ*B which leads to the production of TNF-*α* and IL-6 through TLR3 pathway (*P* < 0.05).

## 4. Discussion

Recent studies have confirmed that many herbs have effects of modulating immunity and inhibiting and killing pathogenic microorganisms [22, 23]. As one of the bioactive ingredients from the natural traditional Chinese medicinal herb* Astragalus membranaceus*, APS is used widely as an immunomodulator in China. Evidences indicate that APS can enhance lymphocyte blastogenesis and stimulate macrophage activation without cytotoxic effects [7, 8]. APS cannot inhibit virus directly but it can activate the immune system and induce the production of cytokines to initiate an antiviral response [24, 25]. Consistent with these previous reports, the current study showed that HSV-1 infection induced the secretion of TNF-*α* and IL-6 in astrocytes and the secretion notably increased in response to APS. These results indicate that APS can promote immunomodulatory effects of astrocytes.

In addition, we also found that HSV-1 infection induced a higher expression of TLR3 and decreased the proliferation of astrocytes; these results are in accordance with previous reports which showed that high expression of TLR3 impaired cell proliferation and inhibited cell cycle progress [26, 27]. Furthermore, we found that APS was capable of promoting the proliferation of astrocytes infected by HSV-1. This result squares with what we already know about the role of APS on cell proliferation [18, 28, 29]. Although the potential mechanism is still to be clarified, regarding the current understanding of TLR3 signaling function, we infer that the effect of APS on proliferation of astrocytes may not be associated with its effect on TLR3.

Antiviral innate immunity depends on different sensor systems that recognize viral-pathogen-associated molecular patterns (PAMPs) and affect specific signaling pathways, including those leading to the activation of NF-*κ*B. Recent studies demonstrate that TLR3 is also present in cells as a sensor to recognize the structure of the pathogens and participate in the adaptive immune response directly [30]. TLR3 can recognize the dsRNA of viruses, initiates intracellular signal transduction pathway, and then induces the activation of NF-*κ*B [30, 31]. Activated NF-*κ*B translocates from cytoplasm to the nucleus, combines with target genes to trigger gene transcriptions, and increases the production of certain antiapoptotic proteins and proinflammatory cytokines [26–28]. Our previous study revealed that when astrocytes were infected by HSV-1, the NF-*κ*B was activated through TLR3 and the generation of TNF-*α* and IL-6 increased obviously [5]. In this paper, we further confirmed that TLR3 and NF-*κ*B positively contribute to the immune response to HSV-1 infection. Moreover, we showed that, for HSV-1 infected astrocytes, APS could promote the expression of TLR3 and the activation of NF-*κ*B which elicits the secretion of inflammatory cytokines TNF-*α* and IL-6, indicating that APS can protect astrocytes by promoting immunological function provoked by HSV-1 through TLR3/NF-*κ*B pathway.

## 5. Conclusion

In conclusion, APS can protect the astrocytes against HSV-1 induced proliferation inhibition and enhance the immunological function of astrocytes by upregulating the TLR3/NF-*κ*B signaling pathway along with increasing expression of TNF-*α* and IL-6. The study suggests that APS has potential in the treatment of HSV-1 infectious diseases in central nervous system.
